# Rapalink-1 Targets Glioblastoma Stem Cells and Acts Synergistically with Tumor Treating Fields to Reduce Resistance against Temozolomide

**DOI:** 10.3390/cancers12123859

**Published:** 2020-12-21

**Authors:** Andres Vargas-Toscano, Ann-Christin Nickel, Guanzhang Li, Marcel Alexander Kamp, Sajjad Muhammad, Gabriel Leprivier, Ellen Fritsche, Roger A. Barker, Michael Sabel, Hans-Jakob Steiger, Wei Zhang, Daniel Hänggi, Ulf Dietrich Kahlert

**Affiliations:** 1 Clinic for Neurosurgery, Medical Faculty, Heinrich-Heine University Düsseldorf, 40225 Düsseldorf, Germany; Andres.VargasToscano@med.uni-duesseldorf.de (A.V.-T.); Ann-Christin.Nickel@med.uni-duesseldorf.de (A.-C.N.); marcelalexander.kamp@med.uni-duesseldorf.de (M.A.K.); sajjad.muhammad@med.uni-duesseldorf.de (S.M.); Michael.Sabel@med.uni-duesseldorf.de (M.S.); Hans-Jakob.Steiger@med.uni-duesseldorf.de (H.-J.S.); daniel.haenggi@med.uni-duesseldorf.de (D.H.); 2 Beijing Neurosurgical Institute, Tiantan Hospital, Beijing 100050, China; liguanzhang122@163.com (G.L.); zhangwei_vincent@126.com (W.Z.); 3 Institute of Neuropathology, Heinrich-Heine University Düsseldorf, 40225 Düsseldorf, Germany; GabrielClementGuillaume.Leprivier@med.uni-duesseldorf.de; 4 Leibniz Research Institute for Environmental Medicine (IUF) and Medical Faculty Heinrich-Heine University Düsseldorf, 40225 Düsseldorf, Germany; ellen.fritsche@iuf-duesseldorf.de; 5 Department of Clinical Neurosciences and WT-MRC Stem Cell Institute, University of Cambridge, Cambridge CB2 1QN, UK; rab46@cam.ac.uk; 6 German Consortium for Translational Cancer Research (DKTK), Essen/Düsseldorf, 40225 Düsseldorf, Germany

**Keywords:** glioblastoma, rapalink-1, tumor treating fields, EMT, therapy resistance, human stem cell in vitro platform, drug development, risk assessment, mTOR

## Abstract

**Simple Summary:**

Glioblastoma (GBM) resistance to standard treatment is driven by stem-like cell behavior and epithelial-like-mesenchymal transition. The main purpose of this paper was to functionally validate a novel discovered pharmacological strategy to treat GBM, the dual mTOR pathway inhibitor Rapalink-1 (RL1) using relevant stem cell models of the disease to unravel mechanistic insights. Our approach also interrogates combination studies with clinical treatment options of tumor treating fields (TTFields) and the best standard of care chemotherapy, temozolomide (TMZ). We provided clinical relevance of our experimental work through in silico evaluation on molecular data of diverse patient samples. RL1 effectively impaired motility and clonogenicity of GBM stem cells and reduced the expression of stem cell molecules. We elucidated a synergistic therapeutic potential of the inhibitor with TTFields to minimize therapy resistance toward TMZ, which supports its consideration for further translational oriented studies.

**Abstract:**

Glioblastoma (GBM) is a lethal disease with limited clinical treatment options available. Recently, a new inhibitor targeting the prominent cancer signaling pathway mTOR was discovered (Rapalink-1), but its therapeutic potential on stem cell populations of GBM is unknown. We applied a collection of physiological relevant organoid-like stem cell models of GBM and studied the effect of RL1 exposure on various cellular features as well as on the expression of mTOR signaling targets and stem cell molecules. We also undertook combination treatments with this agent and clinical GBM treatments tumor treating fields (TTFields) and the standard-of-care drug temozolomide, TMZ. Low nanomolar (nM) RL1 treatment significantly reduced cell growth, proliferation, migration, and clonogenic potential of our stem cell models. It acted synergistically to reduce cell growth when applied in combination with TMZ and TTFields. We performed an in silico analysis from the molecular data of diverse patient samples to probe for a relationship between the expression of mTOR genes, and mesenchymal markers in different GBM cohorts. We supported the in silico results with correlative protein data retrieved from tumor specimens. Our study further validates mTOR signaling as a druggable target in GBM and supports RL1, representing a promising therapeutic target in brain oncology.

## 1. Introduction

Glioblastoma (GBM) is a fatal disease that can occur at any age, with a worse prognosis in older patients. The disease is considered to be driven by glioblastoma stem cells (GSCs), a small subpopulation of highly tumorigenic cells that have stem-like properties including self-renewal, high proliferation rates, and an ability to generate and regenerate different progenies of the tumor [1,2,3,4,5]. GSCs also regulate the molecular process of epithelial-like-to-mesenchymal-like transition (EMT) process, in which the cells acquire a greater motile and therapy resistant phenotype [6].

After maximal safe surgical macroscopic resection of a newly diagnosed GBM (ndGBM), the consensus for best standard-of-care (BSC) involves treating the microscopic dissemination and/or the remaining unresected tumor through a combination of the chemoagent temozolomide (TMZ) and radiotherapy [1,7]. The constitution of the genomic loci of isocitrate dehydrogenase 1 (IDH1) and the activation of O [6]-methylguanine-DNA methyltransferase (MGMT) have been identified to be predictive for BSC response [8,9]. Additionally, sub classification of GBM into transcriptomic or epigenetic subtypes, associated with different gene activation signatures and clinical features have been described [10]; namely, proneural (PN), neural (NE), mesenchymal (MES), and classical (CL). Despite this progress in clinical diagnostics, adequate advances on the therapeutic side, targeting molecular features of the disease, are lagging behind. Anti-stem cell/anti-EMT directed therapies, ideally in a disease subtype specific manner, are desirable [11].

Recently, a novel therapy termed tumor treating fields (TTFields) has been approved for clinical treatment as both monotherapy for recurrent GBM (rGBM) and in combination with adjuvant post-chemoradiation TMZ for ndGBM [12,13]. It is believed to generate a main non-invasive antimitotic effect on the cells by delivering low intensity, intermediate frequency, alternating electric fields, locally targeting the tumor bed. However, the mode of action is not fully understood [14]. Additionally, the available knowledge as to its therapeutic potential in combination with other treatment regimens, or its potential to attack GSC/EMT is not fully known.

Mammalian Target of Rapamycin (mTOR) is a hallmark signaling pathway in cancer including GBM, frequently taken into account as therapeutic targets in major clinical studies [15,16]. Elevated mTOR signaling activity has been associated with the activation of EMT and GSCs maintenance [17,18,19]. Historically, the clinical translation of pharmacological strategies to block mTOR signaling has been challenging due to the emergence of therapy resistance or infectivity in signal suppression under therapy [20,21]. However, the recent generation of mTOR inhibitors directed simultaneously against the two branches of the network (mTORC1 and mTORC2) a.k.a. Rapalinks, have shown great therapeutic potential in an experimental trial of GBM [22]. However, hardly anything is known on the effects of Rapalinks on GSCs EMT biology in GBM.

Thus, we sought to tackle these knowledge gaps by using an in vitro platform composed of well-characterized GSCs and non-cancer neural stem cells derived from fetal origin or human induced pluripotent stem cells. Our drug characterization study employed a wide portfolio of functional bioassays and also included an ex vivo analysis after an in silico interrogation of clinical datasets to further validate the clinical relevance of our experimental findings. By integrating an experimental TTFields system in our experimental design, we believe our results are of interest for translational oriented research community of neuro oncology and beyond.

## 2. Results

### 2.1. Rapalink-1 Inhibits Cell Growth in Glioblastoma Stem Cells (GSCs) Compared to Non-Cancerous Cells

**Figure 1:**
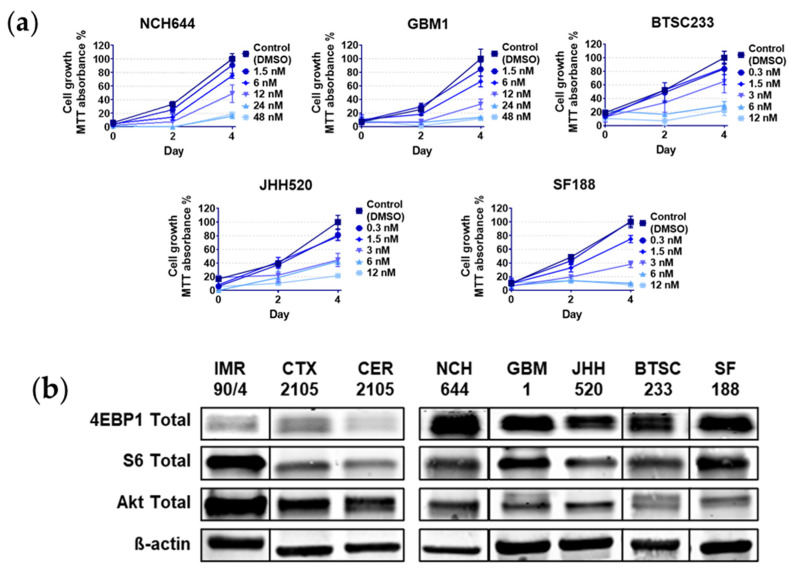

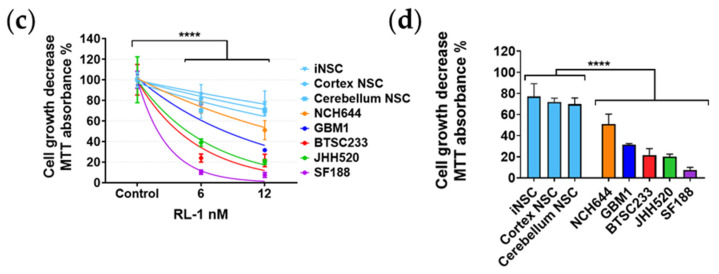
RL1 cell growth effects. (**a**) Cell growth of glioblastoma stem cells (GSCs) measured with MTT (3-(4,5-dimethylthiazol-2-yl) 2,5-diphenyl tetrazolium bromide) absorbance assay after two and four days of incubation, (**b**) main protein expression proteins of the mTOR pathway in cancerous and non-cancerous cells, (**c**) cell growth dose dependent decrease comparison measured with MTT absorbance after 4 days of incubation, (**d**) 4-day incubation significant difference of cell growth decrease measured with MTT absorbance of cancer GSCs compared to non-cancerous NSCs. All the plots present the mean and the standard deviation. The *p*-value < 0.05 was considered statistically significant in all analysis. Statistical tests performed for two variables, unpaired Student’s *t*-test, for more than two related variables, one-way-ANOVA. The significance of the difference between groups was described as **** *p* < 0.0001.

We used established GSC models in order to corroborate the effect of a chosen compound of the Rapalink class (Rapalink-1, RL1) [22] in our models. Details about the molecular properties of the models can be found in Figure 1 and Table 1. The drug dose dependent decrease in cell growth was just visible at two days with a clear effect peak at four days (Figure 1a). We then calculated the respective half maximal inhibitory concentration (IC_50_) values, leading to values in the nano-molar (nM) range (Table 1). In order to have a non-cancerous control for the cell growth analysis, we treated and calculated the IC_50_ from an induced neural stem cell (iNSC) model derived from induced pluripotent stem cells (IMR90/4), and from fetal brain cortex (CTX) and cerebellum (CER) derived neural stem cells. We then compared the main protein expression profiles of the mTOR pathway, namely eukaryotic translation initiation factor 4E-binding protein 1 (4EBP1), ribosomal protein S6 (S6), and Akt with a clearly higher mTOR expression in cancer cells compared to non-cancerous stem cells, mainly with the 4EBP1 marker (Figure 1b). Based on curve analysis and IC_50_ values, the cell line NCH644, characterized as PN [23], was the most resistant cancerous model, followed by the CL-subtype cell line GBM1 [24]. The MES subtypes BTSC233 and JHH520 [25,26] followed in drug sensitivity, finally followed by the pediatric glioblastoma model SF188, which was the most sensitive and only MGMT-unmethylated cell line used (Figure 1c,d). All cell lines were classified accordingly by other institutions and verified by our lab using RNA sequencing data from an ongoing project [10,27]. Furthermore, the effect of RL1 was significantly more potent in cancerous models compared to the non-cancerous tested models (Figure 1d).

### 2.2. RL1 Inhibits mTOR Pathway Signaling Activity

**Figure 2:**
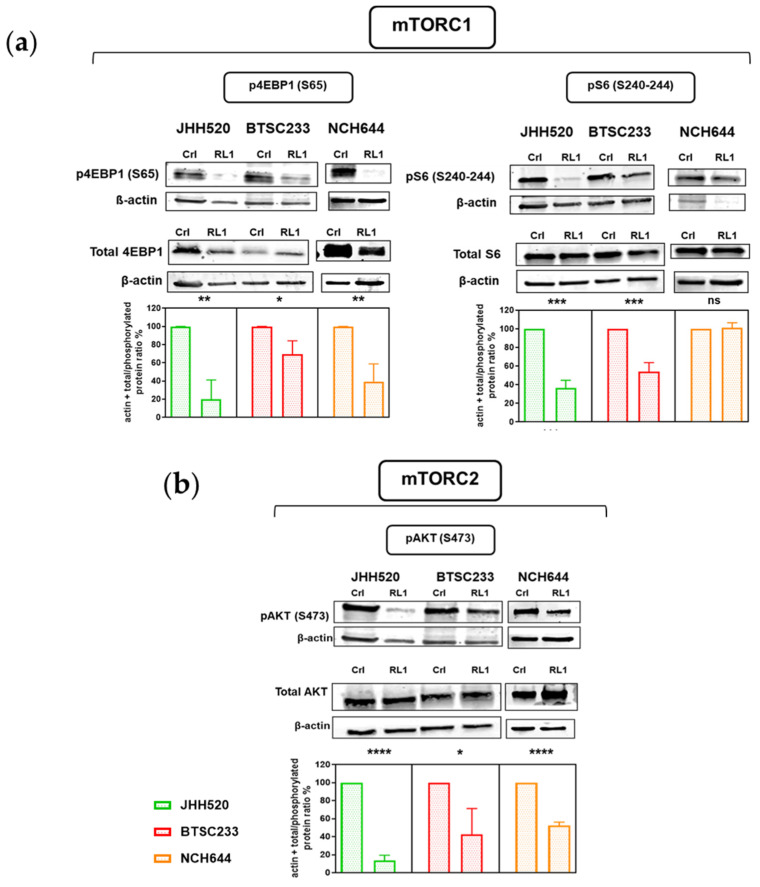
RL1 effect on mTOR signaling protein marker expression. RL1 protein expression analysis validates the inhibition of both (**a**) mTORC1 and (**b**) mTORC2 in our GSCs. Statistical tests performed for two variables with the unpaired Student’s t-test. The significance of the difference between groups was described as * *p* < 0.05, ** *p* < 0.01, *** *p* < 0.001, **** *p* < 0.0001.

Next, we undertook a protein expression analysis to validate if the described effect of RL1 on mTORC1/2 applied to our GSCs (Figure 2a,b). For mTORC1 activity, we used the downstream markers phospho-4EBP1-Ser^65^ and phospho-S6-Ser^473^, and for mTORC2, we used phospho–AKT-Ser^473^. Phosphorylation of most of the proteins used to quantify signaling pathway activity was inhibited by RL1, only the phosphorylated S6 marker was not inhibited in NCH644 (Figure 2a,b). We thus confirmed a dual inhibition of RL1 in the mTORC1 and mTORC2 downstream pathway markers in GSCs.

### 2.3. RL1 Induces Cell Cycle Arrest, Apoptosis, and Proliferation Inhibition

**Figure 3:**
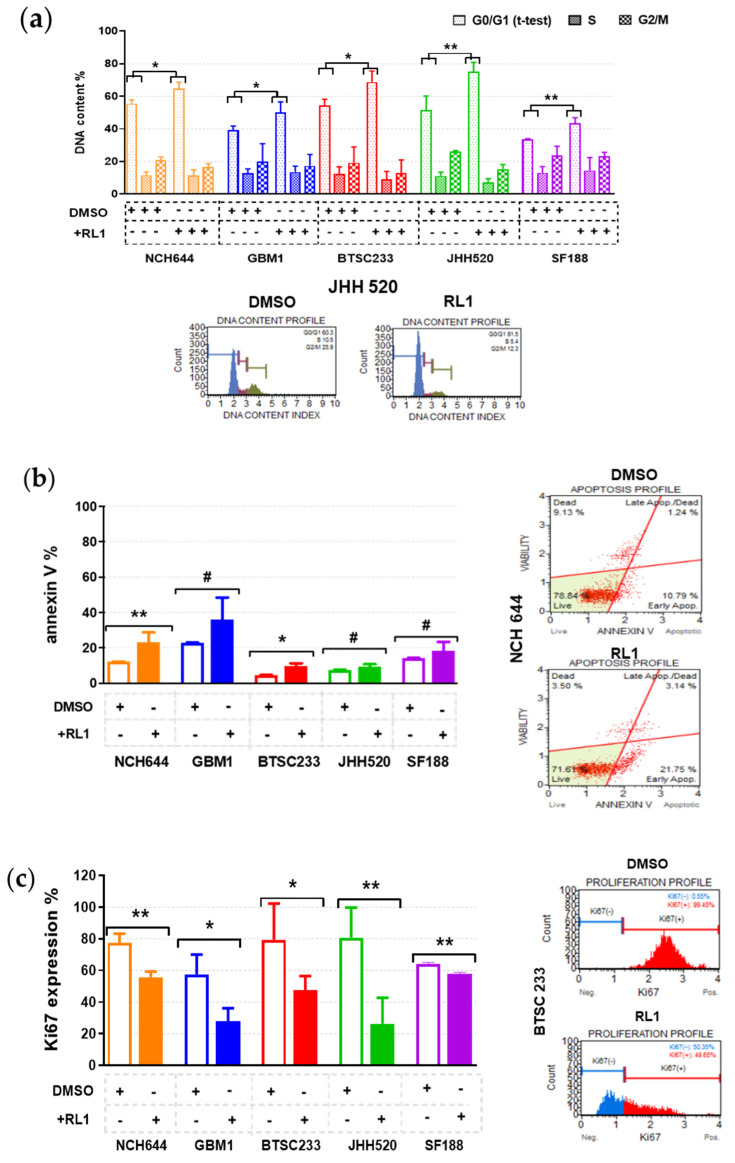
RL1 mechanistic effects. (**a**) Cell cycle arrest in the G0/G1 phase of all the models given DNA content %, (**b**) small apoptosis increase in all cell lines only statistically significant in NCH644 and BTSC233, with numerical increase in the other cell lines, (**c**) significant decrease in GSC proliferation given by Ki67% expression. Statistical tests performed for two variables with the unpaired Student’s *t*-test. The significance of the difference between groups was described as * *p* < 0.05, ** *p* < 0.01, # numerical-nonsignificant.

After determining that the cell growth and mTOR pathway inhibition capacity of RL1 extends to GSCs, we aimed to further characterize the mode of action of this therapy. There was a significant cell cycle arrest in the G0/G1 phase of all models (Figure 3a), corroborating a clear antimitotic effect.

In parallel, there was a slight increase of apoptosis in all the cell lines as an additional effect, but this was only statistically significant in the NCH644 and BTSC233 lines (Figure 3b). In line with this, there was a significant decrease in proliferation for all the tested cell lines (Figure 3c).

### 2.4. RL1 Inhibits Stemness and EMT

**Figure 4:**
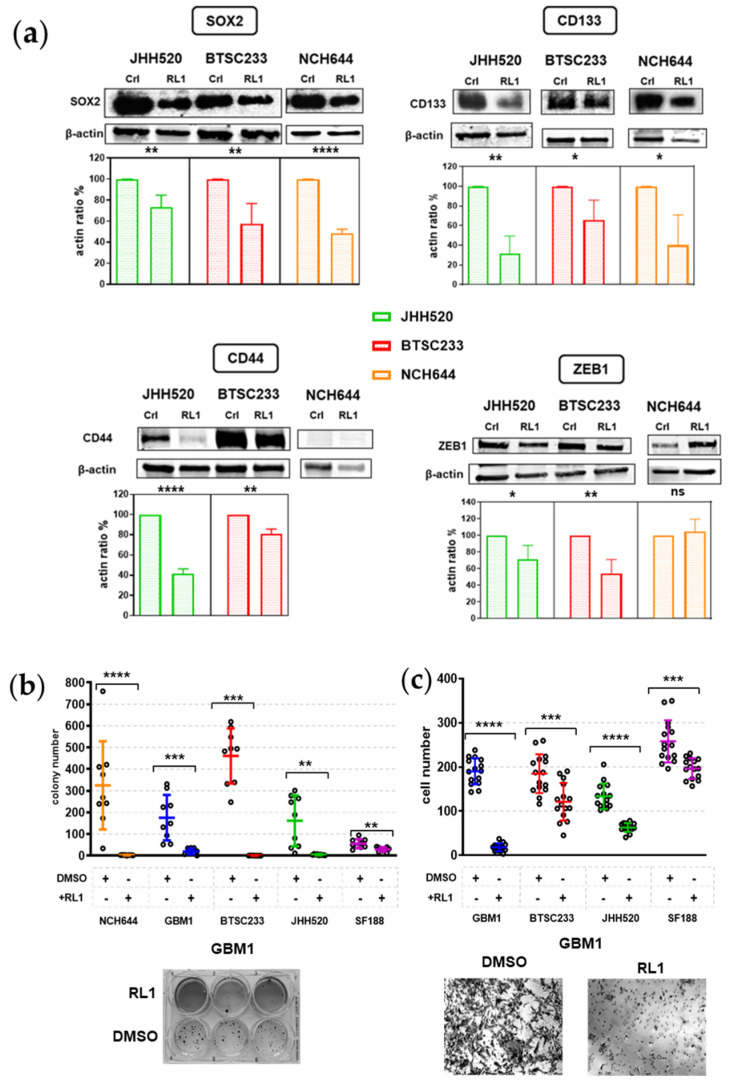
RL1 effect on stemness and EMT protein marker expression. (**a**) RL1 protein expression analysis validates the inhibition of neural stem cell markers and mesenchymal transformation markers in our GSCs, (**b**) RL1 strongly inhibited colony formation in all GSCs in agar assays, (**c**) RL1 strongly inhibited migration in all cell lines except NCH644 in Boyden chamber assays. Statistical tests performed for two variables, unpaired Student’s *t*-test. The significance of the difference between groups was described as * *p* < 0.05, ** *p* < 0.01, *** *p* < 0.001, **** *p* < 0.0001.

Since we identified a wide functional effect of RL1 on our GSCs, we sought to probe for the effects on markers indicating stem cell properties. We chose the validated neural stem cell markers, CD133 and SOX2, and the mesenchymal transformation markers CD44 and ZEB1 and quantified their total protein abundancy. We could not observe all markers in all of our models. CD133 and SOX2 were suppressed by RL1 in NCH644, BTSC233, and JHH520; while CD44 expression was reduced by the same drug in MES BTSC233 and JHH520, the only cell models that were found positive for this protein (Figure 4a). Phenotypically, the ability to form GSC colonies was strongly and significantly inhibited in all cell lines by RL1 (Figure 4b). The master EMT transcription-factor marker ZEB1 was inhibited by RL1 in the BTSC233 and JHH520 MES-type models, but not in PN NCH644 (Figure 4a). The indication of suppressed EMT was phenotypically supported by the fact that RL1 treatment strongly inhibited cellular migration in all cell lines except NCH644, which after several attempts continued to form consolidated neuro-spheres before undergoing migration (Figure 4c).

### 2.5. Association between mTOR Biomarkers and EMT/Mesenchymal Markers Biosamples of GBM Patients of Western and Eastern Ethnicity

**Figure 5:**
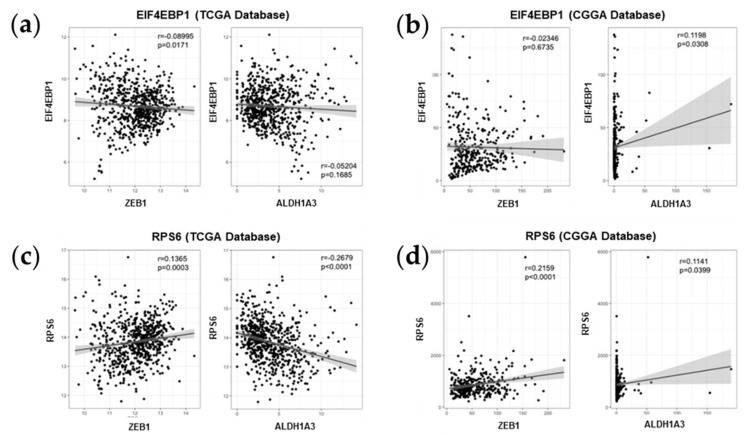

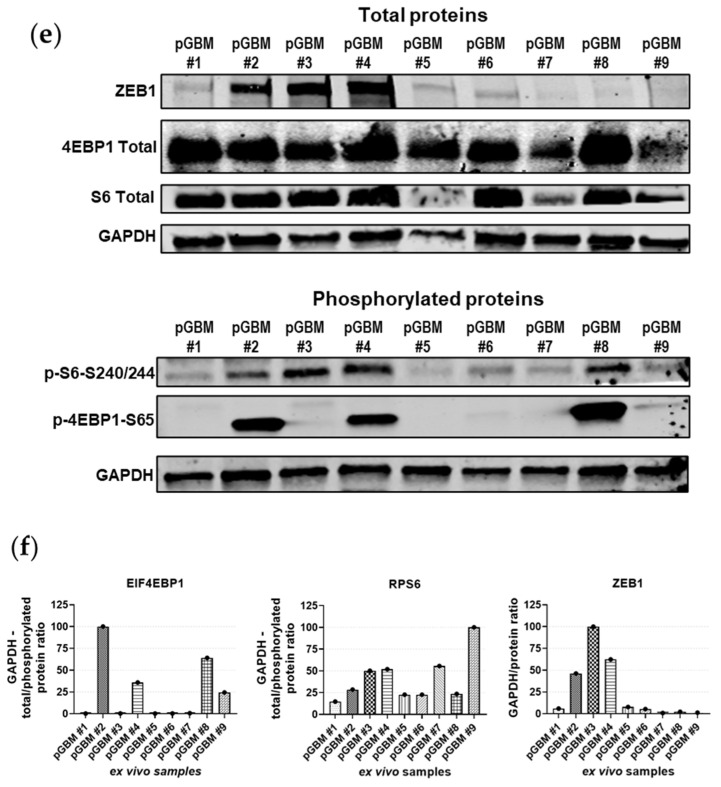
Bio-informatic in silico analysis. (**a**) TCGA *EIF4EBP1* mRNA data, (**b**) CGGA *EIF4EBP1* mRNA data, (**c**) TCGA *RPS6* mRNA data, (**d**) CGGA *RPS6* mRNA data, (**a**–**d**) the graphs were organized from left to right as *ZEB1* and *ALDH1A3*, (**e**) protein expression on surgical samples, (**f**) all proteins are normalized to loading control (GADPH), for EIF4EBP1 and RPS6 the ratio between total protein and phosphorylated form are calculated. The significance of the difference between the groups of data was analyzed by unpaired T test.

To probe for clinical relevance of our experimental data, we performed a data-mining analysis from established clinical cancer sample datasets. To this aim, we used an American cohort (The Cancer Genome Atlas, TCGA) and a Chinese cohort (Chinese Glioma Genome Atlas, CGGA) of patients. In our analysis, we put special emphasis on probing the potential correlation between the expression of two main mTOR signaling genes, namely *EIF4EBP1* (gene encoding 4EBP1) and *RPS6* (gene encoding S6), and one EMT marker, namely *ZEB1* as well as one mesenchymal marker, namely *ALDH1A3*, a recently identified marker for mesenchymal transformation in GBM [28]. We found that while *EIF4EBP1* and *ZEB1* expression were negatively correlated, even though minimally, in the American cohort, this was not observed in the Chinese dataset (Figure 5a,b). There was a significant positive correlation of *EIF4EBP1* and *ALDH1A3* mRNA expression levels in samples from Chinese patients, but not in the American dataset (Figure 5a,b). When analyzing the expression levels of *RPS6* in the same datasets, we found *RPS6* and *ZEB1* mRNA expression levels to be positively correlated in both cohorts (Figure 5c,d). While the expression levels of *ALDH1A3* was negatively correlated with *RPS6* in the American cohort, it was positively correlated with *RPS6* expression in samples from Chinese patients (Figure 5c,d).

Moreover, we verified our in silico analysis with primary GBM samples (pGBM) derived from surgical subjects of our institution. We tested the same relevant markers above-mentioned. We confirmed a high expression of the mTOR signaling pathway molecules in all of the samples. As for the phosphorylation defined proteins, the RPS6 marker was widely activated, in contrast to EIF4EBP1, which was clearly phosphorylated in four samples (pGBM#2, pGBM#4, pGBM#8, pGBM#9). Correlating ZEB1 activity, the samples pGBM#2 and pGBM#4 featuring the highest ZEB1 activity showed a clear correlation of ZEB1 with mTOR activity, whereas pGBM#1, pGBM#5, and pGBM#6 had a mild correlation between mTOR activity and EMT.

### 2.6. TTFields, RL1, and TMZ Synergistically Reduced Cell Growth

**Figure 6:**
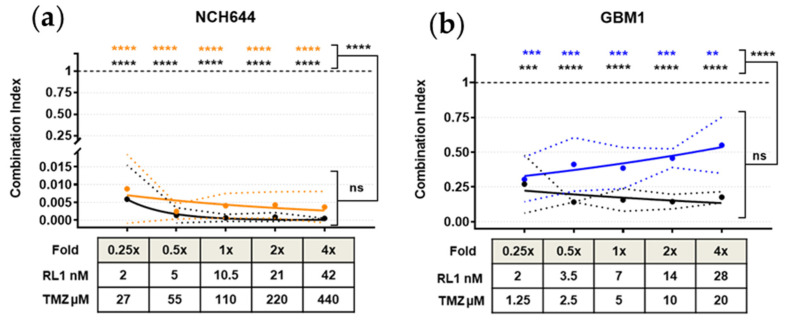

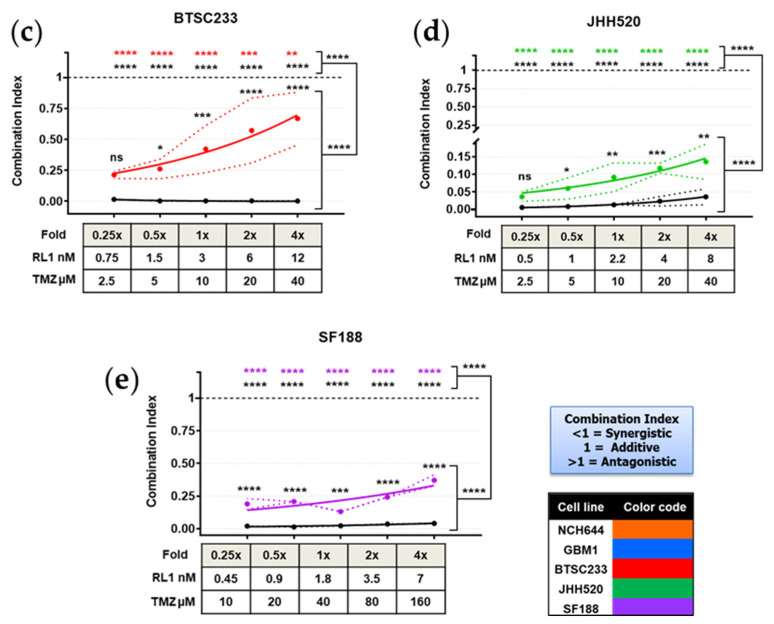
Synergistic effect of RL1, TMZ, and TTFields. (**a**–**e**) All color coded GSCs according to RL1 IC_50_ value showed a significant synergistic effect when combining RL1 with TMZ (colored lines), and an increased significant synergistic effect when adding TTFields to the combination. Under each figure, there is a small table with the multiplication fold values of RL1 and TMZ. The statistical test performed was one-way-ANOVA. The significance of the difference between groups was described as * *p* < 0.05, ** *p* < 0.01, *** *p* < 0.001, **** *p* < 0.0001.

Finally, given the recent advances in the clinic in treating GBM [7,13,28], we included experimental TTFields therapy and TMZ treatment in our study. To identify the IC_50_ of TMZ of our models, we performed the cell growth assays under a range of TMZ treatment concentrations for up to six days (Table 1, Figure 6, Figure S1). This data correlates well with previous literature reports and MGMT promoter methylation characteristics of the respective models with SF188 (the only model with unmethylated MGMT promoter status) having the second lowest IC_50_. The fast ability to form spheres may have increased the treatment resistance of the NCH644 model, which had the lowest IC_50_.

For TTFields, we applied the clinically relevant field frequency of 200 kHz. With our setup, we thus achieved a field intensity of 1.7 V/cm RMS. We then assessed growth on cells under different treatment conditions to probe for any combinational effects when combining RL1 with the clinical treatment scenarios using the Chou–Talalay method [29]. Our treatment setup can be found in Table 2.

A 48-h incubation was an adequate time frame to induce a synergistic dose dependent effect in all the cell lines and groups, except for BTSC233, which required 96 h of incubation in order to show significant synergistic combination index (CI) values -CI < 1 referring to synergism, CI = 1 to additive effect and CI > 1 to antagonism-. NCH644, which was up to this point the most resistant GSC against both RL1 and TMZ, showed the most potent synergistic effect CI < 0.02 *p* < 0.0001 with a tendency of increased CI proportional to the drug concentrations applied, as opposed to the rest of the cell lines, which showed opposite curve directions. Despite this effect, the additional TTFields treatment showed a numerically enhanced, non-significant synergistic CI effect. The cell line JHH520, showed the second most potent synergistic effect of TMZ and RL1 with values CI < 0.2 *p* < 0.0001, which was significantly strengthened by the TTFields treatment. The cell line GBM1 had a more discrete but significant drug combination synergy with CI values lower than 0.75 *p* < 0.0001, however, TTFields treatment showed a numerical, non-significantly stronger synergistic CI effect. For the other cell lines, namely SF188 (CI values < 0.5) and BTSC233 (CI values < 0.9), the synergistic effect was significant, both for the drugs alone (*p* < 0.0001) and stronger with the addition of TTFields treatment (*p* < 0.0001). Our data strongly indicate a synergistic therapeutic potential for RL1 on GSCs when combined with TTFields and TMZ. The TTFields synergy bioassay results showing synergism can be found in Figure 6a–e.

## 3. Discussion

mTOR is a key player in the activation of cell growth, reprogramming of cell metabolism, and structural cytoskeleton remodeling, amongst many others [30]. In the context of cancer, many projects have been conducted dedicated to developing inhibition strategies to effectively block the activation of mTOR signaling activity [31], with some promising clinical trials underway [32]. Additionally, in the context of brain tumors, targeting mTOR activation is considered a potent therapeutic avenue [15,33]. However, given the complex nature of this signaling pathway comprising two molecular distinct signaling branches, enabling the compensation of signal loss from either of the two [30], it is generally accepted that clinically relevant anti-mTOR directed therapies will have to block the entire pathway [34] to avoid the emergence of therapy resistance [31].

In this regard, recent development in campaigns aimed to generate dual mTOR complex inhibitors [22,35] and their functional validation in experimental trials has raised hopes in advancing our ability to treat lethal cancers. By choosing the most promising last generation mTOR inhibitor drug candidate, termed Rapalink1, our study sought to validate its effects on state-of-the-art disease models of the disease. Rapalink1 has previously been shown to effectively penetrate the blood brain barrier and brain parenchyma of the rodent model of brain tumor [22]. We now extend the evidence of the potential of this drug candidate to possess effective anti-cancer stem cell effects and to potentiate clinical approved treatments, at least in vitro. Although we applied 3D organoid-like in vitro models of the disease, future animal studies, especially incorporating the animal setup of TTF, are required to unequivocally postulate the therapeutic relevance of our findings. Of note, our initial off target characterization assay using non cancer cell models indicated that RL1 possesses a higher therapeutic index on cancer cells, supporting this drug substance for further oncology studies.

Our results clearly demonstrated the potency of this drug candidate to be able block stem cell markers and properties including migration and clonogenicity in GBM. The soft agar assay was chosen because of our group’s previous experience using reduction of sphere formation as well as the reduction of protein abundancy of stem cell markers such as ZEB1, as a biomarker combination to indicate the blockade of stem cell phenotype in our disease models [36,37]. Interestingly, we found that the strongest drug effects were seen in the most aggressive cell models of the MES subtype, a subtype with the worst clinical prognosis for overall survival of the patients [38], and more moderate effects in the PN cell model, further advocating this drug candidate to be particularly useful for targeting the highest malignant cell population in GBM. Concordantly, the correlations of mTOR signaling regulating cell cycle [39,40], stemness/EMT [18,30,41], and cellular survival [42] are generally accepted and our results are well in line with those high-profile papers. Thus, together with the translational focus of our study to test the promising drug candidate in a clinical-near experimental setting, our data proved solid ground for mode of action characterization of RL1 on GSCs featuring efficient target suppression, consequently blocking stemness/EMT.

Next, we performed a confirmatory in silico study to probe for the clinical relevance of mTOR and EMT biomarkers in large scale molecular datasets of clinical samples from cohorts of different genders and ethnicities. We identified a tendency of direct correlation between the expression of the mTOR gene *RPS6* and the expression of the mesenchymal marker *ZEB1* in two different cohorts (TCGA and CGGA cohorts), suggesting a link between the mTOR pathway and EMT in GBM patient samples. This is in line with a previous study identifying the mTOR pathway as a prognostic gene set in the MES subtype [43]. The results of our drug validation and patient sample studies are in line with the work of others that have identified mTOR signaling as a promoter of EMT and stemness in various diseases as well as in normal development [19]. Furthermore, previous recent work of others have already identified the existence of an mTOR-ZEB1 signaling axis in GBM using various functional attempts, which we now confirm in the context of a therapeutic relevant pharmacological in vitro model [44,45,46]. Together with our correlative assays of transcript and protein abundancy in patient samples, we hypothesize that the mTOR-ZEB1 axis extends to GSCs, but seems differentially aberrant amongst individual GBM cases. However, in all tested cases, RL1 provides an efficient option to effectively reduce their activation, leading to desired anti-cancer cell effects.

After corroborating an antimitotic and partially apoptotic effect of RL1 on GSCs, known to be highly resistant to standard clinical treatments [47], we wanted to see if RL1 can augment the effectivity of clinical GBM treatments. We executed detailed combination treatment studies in vitro featuring RL1, BSC chemotherapeutic agent TMZ as well as TTFields. The widely used drug combination effect method described by Chou and Talalay [29] was chosen to guide our experimental design and the quantification of results to identify any potentiating effects. Applying inovitro™ settings that mimic the TTFields therapy used in the clinical setting, we found a synergistic anti-cell growth effect when combining RL1 with TMZ and TTFields. Follow up testing in vivo, using the recently launched animal system for TTFields, inovivo™, will now need to be done to validate the therapeutic potential of this treatment regime. Nevertheless, given the previously described therapeutic effect of RL1 in animal models of human GBM [22], we believe that our results already support the consideration of this treatment option from a clinician-scientist point of view.

It was demonstrated that the binding of RL1 to the factor termed FKBP12 is required to inhibit mTOR activity and to mediate the anti-proliferative effect of Rapalink1 [22,48]. Therefore, it is highly likely that the effect of Rapalink1 on stemness and EMT markers as well as its synergy with tumor treating fields requires FKBP12. Since FKBP12 has not been proven as a clinically relevant marker, this may be a relevant approach for further studies.

In summary, we used our diverse stem cell in vitro platform to perform mode of action analysis and initial risk assessment of a novel drug candidate, and interrogated clinical treatment options to benchmark its therapeutic potential in combination regimes. We used clinical specimens from ethnic and gender diverse backgrounds to validate our experimental findings to benchmark relevance.

## 4. Materials and Methods

### 4.1. Cell Culture, Fresh Patient Samples, and Pharmacologic Substances

The in vitro cell models used were kindly provided as follows, glioblastoma neuro-spheres JHH520 (G. Riggins, Johns Hopkins, Baltimore, MD, USA); SF188 (E. Raabe, Johns Hopkins, Baltimore, MA, USA); BTSC233 (M.S. Carro, Freiburg University, Freiburg im Breisgau, Germany); NCH644 (C. Herold-Mende, Heidelberg University, Heidelberg, Germany); and GBM1 (A. Vescovi, San Raffaele Hospital, Milano, Italy). Ethical approval for the use of cell models to study brain cancer biology was from the ethical commission of the medical faculty of Heinrich-Heine University (study ID 5841R). Cortical fetal neural stem cells were collected from human fetal cortical tissue grown in a neurosphere condition (ethical vote Study ID #5206). Induced neural stem cells were differentiated from the human iPSC line IMR 90/4 (WiCell, Madison, WI, USA), as previously described [49]. 

The primary GBM tumor samples were derived from the operation room of the department of neurosurgery (Düsseldorf, Germany) and were snap-frozen in liquid nitrogen until the preparation of lysates. All subjects gave their informed consent for inclusion before their participation in the study. The study was conducted in accordance with the Declaration of Helsinki, and the protocol was approved by the Ethics Committee of the Medical Faculty of the Heinrich-Heine University of Duesseldorf (#2019-484-FmB). 

All cells were grown in complete serum-free suspension media enriched with bovine fibroblast growth factor (Peprotech, Rocky Hill, NJ, USA) and human epidermal growth factor (Peprotech), as previously described [50]. These were incubated under standard conditions (SCs, humidified 37 °C, 5% carbon dioxide (CO_2_)). Cells were regularly tested for mycoplasma accumulation and authenticity using the short tandem repeat assay, as previously described [51]. Rapalink-1 was purchased from Apexbio Technology (Houston, TX USA) and TMZ (Sigma-Aldrich, St. Louis, MO, USA). Both were resuspended in a DMSO (dimethyl sulfoxide, Sigma-Aldrich) vehicle, according to their molecular weight and the manufacturer’s instructions, after which we further diluted them to the required concentrations and stored them at −20 °C.

### 4.2. Cell Growth (MTT Assay)

Cell growth of the different models was assessed using the MTT (3-(4,5-dimethylthiazol-2-yl) 2,5-diphenyl tetrazolium bromide; Sigma Aldrich) assay and plating 3000 cells per well in technical triplicates of 100 µL growth-media each in clear-96-well plates (Corning Inc., Corning, NY, USA). On each plate, we included a respective cell control triplicate (media, cells, and DMSO < 1%) and a blank control (media, no cells) to normalize the cell growth and background reading. We tested RL1 at the following concentrations of 1.5, 6, 12, 24, and 48 nM. For TMZ, 5, 10, 25, 50, and 100 µM. Cells were then incubated with RL1, initially over six days, then finally over four days in SCs. In parallel, for TMZ, this was done for six days in SCs. Starting from day 0 and then every other day (days 2, 4, or 6), we measured the MTT absorbance values as follows: we added 10% of MTT reagent per well, incubated the cells in SCs for three hours, verified the formation of crystals under bright field microscope, and finally lysed the cells by incubating them with HCl-isopropanol-TritonX for 10 min. The resulting relative absorbance was finally measured with the Paradigm micro-plate reader (Molecular Devices LLC, San Jose, CA, USA). All experiments were done in three independent biological repetitions before statistical analysis and IC_50_ calculations.

### 4.3. Tumor Treating Fields

The inovitro™ preclinical laboratory research system dishes (Novocure, Saint Helier, Jersey) were plated in parallel to the 35 mm cell culture dishes and similarly contained 40,000 cells in 2 mL of complete media per plate. Using the previously calculated IC_50_; different multiplication folds of drug concentrations were applied to each condition in the inovitro™ dishes as follows:

- IC_50_ fold of RL1 (0.25×, 0.5×, 1×, 2×, 4×) plus DMSO control.
- IC_50_ fold of TMZ (0.25, 0.5, 1, 2, 4) plus DMSO control.
- Combined IC_50_ folds of TMZ and RL1 in ascending order (0.25 + 0.25, 0.5 + 0.5, 1 + 1, 2 + 2, 4 + 4) plus DMSO control.

Immediately after, they were treated with TTFields (1.7 V/cm RMS) via the inovitro™ system by using perpendicular pairs of transducers insulated by a high dielectric constant ceramic. TTFields were applied for 48 h (except BTSC233 with 96-h treatment) at a frequency of 200 kHz, the optimal frequency established clinically for glioblastoma patients at final temperature of 37 °C, in a humidified incubator with 5% CO_2_.

In parallel, identically plated non-TTFields control 35 mm cell culture dishes were incubated in SCs. All dishes/plates/conditions had an equal DMSO concentration of less than 1%.

After 48 h of treatment or incubation, the different cell lines were re-plated in clear 96-well-plates and processed as described above for the MTT assays. All experiments were done using three independent biological repetitions before statistical analysis.

### 4.4. Synergy Assays

The CI, based on the unified theory of the median effect equation, was calculated using the Chou method, as previously described [36,52] using the CompuSyn Software^®^ (ComboSyn Inc., Paramus, NJ, USA). The software algorithm defined as CI < 1 referring to synergism, CI = 1 to additive effect, and CI > 1 to antagonism.

The significance of the synergistic effect of all treatments was calculated compared to the additive effect (CI = 1), using the resulting CI values of each triplicate. The closer to CI = 0, the stronger the synergy, and the closer to CI = 1 or >1, the lower the synergism. Using this, the significance of the RL1 effect compared to TMZ was calculated comparing the resulting CI values of each combination.

### 4.5. Flow Cytometry–Muse© Assays

Using the Muse © Cell Analyzer Flow Cytometer device (Merck, Darmstadt, Germany) as previously described [53], we tested apoptosis assays with the Annexin V & Dead Cell Kit (Luminex, TX, USA), proliferation assays with the Ki67 Proliferation Kit (Luminex), and the cell cycle assay with the Cell Cycle Kit (Luminex), according to the manufacturer’s protocols. All kits used a 7aad fluorophore marker. All assays were performed after 48 h, in order to have an earlier drug effect incubation, with the previously calculated IC_50_ drug concentration, in independent biological triplicates.

### 4.6. Migration–Boyden Chamber Assay

Assessment of the cellular migration was performed using a modified 24-well Boyden Chamber assay similar to the invasion assay described before [50], but without coating, in order to determine the migration effect. 75,000 cells were suspended in 500 mL of DMEM and placed on top of each insert membrane (Life Technologies, Carlsberg, CA, USA). The bottom was filled with 700 mL DMEM media containing 10% fetal calf serum. All Boyden chamber assays were analyzed 14 h after cell plating. The upper side of the membrane was then wiped with a moist PBS (phosphate-buffered saline) cotton swab to remove the remaining plated cells. The membrane was then fixed at −20 °C with methanol for 15 min and stained with hematoxylin. The invasion of the cells was evaluated by counting the cell nuclei on the lower side of the membrane under a bright field microscope, counting five random high-power fields per insert in three independent biological repetitions. The migrated cells were quantified using ImageJ software (National Institutes of Health, Maryland, USA), before statistical analysis.

### 4.7. Clonogenicity–Colony Formation Assay

To evaluate the clonogenic capacity of our cell models, we performed a colony formation assay in soft agarose as described previously [37]. Clear six-well plates (Corning Inc.) were initially coated with a bottom layer of 1.5 mL of 1% agarose (Life Technologies) and complete media, then incubated for at least 1 h at room temperature. Afterward, a 2 mL layer of 0.6% agarose with 5000 cell per well was plated for all cells except SF188, which required 10,000 cells per well to generate clear clones. It was then covered with an additional 2 mL of fresh media, which was changed every three days. After three weeks, colonies were stained with 1 mg/mL 4-nitro blue tetrazolium chloride (NBT) solution (Sigma-Aldrich), and incubated overnight in SCs; three independent biological repetitions were performed before the colonies were quantified using Clono-Counter software [54], and the statistical analysis was performed. This assay was established to functionally verify the stem cell properties of our cell models [37,55].

### 4.8. Protein Expression–Western Blot

Western blotting was done as previously described [50]; antibodies were used as per the manufacturer’s instructions (for specifications, see Table S1 and Figure S2. Western blots membranes). The total protein content of each cell line was extracted using RIPA Buffer, then determined colorimetrically using the DC Protein Assay Kit (Bio-Rad, Hercules, CA, USA), according to the manufacturer’s instructions, and measured with the Paradigm micro-plate reader. Primary antibodies (as reported in Supplementary Table S1) were incubated overnight at 4° on a rocking platform. Secondary antibodies (goat-anti-rabbit, IRDye800CW LI-COR #926-32211; goat-anti-mouse, IRDye680RD LI-COR #926-68070; goat anti-rabbit-HRP, Jackson Immuno Research #111-035-144; all 1/10,000) were incubated for 1 h at room temperature. All antibodies were diluted in blocking solution containing either 5% bovine serum albumin (BSA) for phosphorylated proteins, or 5% milk powder for the rest of the non-phosphorylated proteins; both diluted in Tris-buffered saline with Tween20 (TBST). For the phospho-proteins, we used a BSA blocking agent that allowed clear bands, since albumin tends to not be phosphorylated; and we normalized the resulting inhibited proteins with the total non-phosphorylated corresponding mTORC1 and mTORC2 markers. Signals were detected using either a film-based system by applying a Super Signal West Pico Chemiluminescent Substrate (Thermo Scientific) or a luminescence-based system in a LI-COR Odyssey CLx Imager (LI-COR). Densitometry quantification was done either with the supplied software from LI-COR or ImageJ software for the films. Experiments were performed using three independent biological repetitions before statistical analysis.

### 4.9. Bio-Informatic Analysis

Transcriptome sequencing data and clinical data of glioma patients were obtained from the CGGA (Chinese Glioma Genome Atlas) database (https://www.cgga.org.cn) and TCGA (The Cancer Genome Atlas Program) database (https://tcgadata.nci.nih.gov). For stemness, we tested the ALDH1A3 in order to complement the limited reports of this marker in the published literature. For EMT, we explored the master transcription factor ZEB1. The statistical computations and figure drawing were performed with R package ‘ggplot2’.

The data used from CGGA were approved by the Beijing Tiantan Hospital Institutional Review Board and tumor specimen quality control.

### 4.10. Statistical Analysis

All graphs and analyses were calculated with Prism GraphPad 8 software (San Diego, CA, USA) except for the bioinformatics analysis, which was performed with the R package above-mentioned. The media control absorbance value average was rested from all wells. The average absorbance value of every DMSO-vehicle-control cell containing wells was used as the control for normalization. IC_50_ values were calculated using a logarithmic nonlinear regression formula in the aforementioned software. The performed statistical tests depended on the related variables; for two variables, the unpaired Student’s *t*-test, and for more than two related variables, one-way-ANOVA was applied. All plots present the mean and standard deviation. The *p*-value < 0.05 was considered statistically significant in all analysis. The significance of the difference between groups was described as * *p* < 0.05, ** *p* < 0.01, *** *p* < 0.001, **** *p* < 0.0001.

## 5. Conclusions

We validated the therapeutic potential of RL1 against GBM using advanced human stem cell disease modeling technology and identified its synergistic effect potency when combined with TTFields and TMZ, two of the main clinically approved treatment options for this disease. By showing fewer toxic effects on non-cancer stem cells, we validated our platform technology to be of benefit for drug development and for projects that assess the risk of substances applied in experimental or clinical contexts. Given the previous report on the effectiveness of RL1 in animal models of human GBM, our results support clinical trials of RL1 in patients with GBM.

## Figures and Tables

**Table 1:** Cell line characteristics.

Type	Cell Line	Color Code ^1^	RL-1 IC_50_ nM	TMZ IC_50_ µM	Gender	Age Group	Molecular Subtype (Verhaak)	MGMT Status	IDH Status	ALDH1A3 Expression
Glioblastoma stem cells	NCH644		10.5	110	Female	Adult	Proneural	Methylated	Wildtype	Negative
	GBM1		7	5	Male	Adult	Classical	Methylated	Wildtype	Positive
	BTSC233		3	10	Female	Adult	Mesenchymal	Methylated	Wildtype	Positive
	JHH520		2.2	10	Female	Adult	Mesenchymal	Methylated	Wildtype	Positive
	SF188		1.8	40	Male	Pediatric	-	Unmethylated	Wildtype	Positive
Induced Neural Stem Cell	IMR 90/4		15.5	-	-	-	-	-	-	-
Neural Stem Cells	Cortex		12	-	-	-	-	-	-	-
	Cerebellum		11	-	-	-	-	-	-	-

^1^ Color code defined to simplify the interpretation of the figures.

**Table 2:** Experimental setup for combination treatment study.

Dish Configuration	Value	Drug	Concentration
Frequency	200 kHz	IC_50_ folds of RL1	0.25×, 0.5×, 1×, 2×, 4× Plus DMSO control.
Temperature	37 °C		
Current	12 mA	IC_50_ folds of TMZ	0.25×, 0.5×, 1×, 2×, 4× Plus DMSO control.
Voltage	1.7 V/cm RMS	Combined IC_50_ folds of TMZ and RL1 in ascending order	0.25 + 0.25, 0.5 + 0.5, 1 + 1, 2 + 2, 4 + 4 Plus DMSO control.
Incubation Time	All 48 h BTSC233 96 h		

## Supplementary Materials

The following are available online at https://www.mdpi.com/2072-6694/12/12/3859/s1, Figure S1: TMZ cell growth and IC_50_; Figure S2: Western blots membranes. Table S1: Western blot antibody concentrations.
