# Supplementary material for: Rapalink-1 Targets Glioblastoma Stem Cells and Acts Synergistically with Tumor Treating Fields to Reduce Resistance against Temozolomide
Source: Cancers (Basel). 2020 Dec 21;12(12):3859. doi: 10.3390/cancers12123859 (PMC7766508; doi:10.3390/cancers12123859)
Supplement: Supplementary file 1 [file cancers-12-03859-s001.pdf]

Rapalink-1 Targets Glioblastoma Stem Cells and Acts Synergistically with Tumor Treating Fields to Reduce Resistance against Temozolomide

Andres Vargas-Toscano, Ann-Christin Nickel, Guanzhang Li, Marcel Alexander Kamp, Sajjad Muhammad, Gabriel Leprivier, Ellen Fritsche, Roger A. Barker, Michael Sabel, Hans-Jakob Steiger, Wei Zhang, Daniel Hänggi and Ulf Dietrich Kahlert

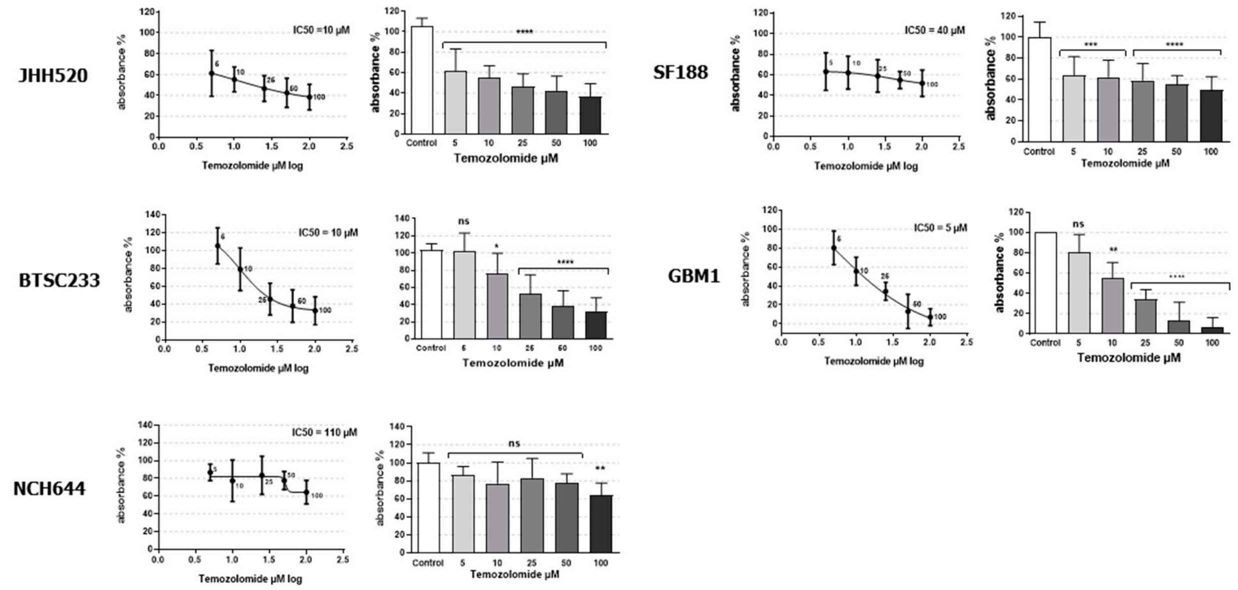

Figure S1. TMZ cell growth and IC50.

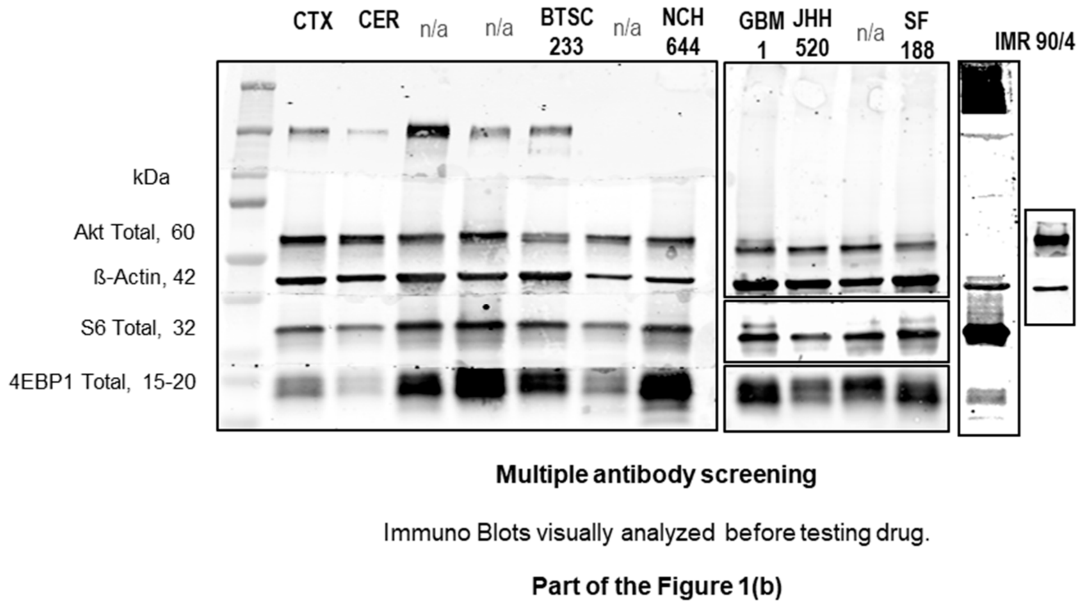

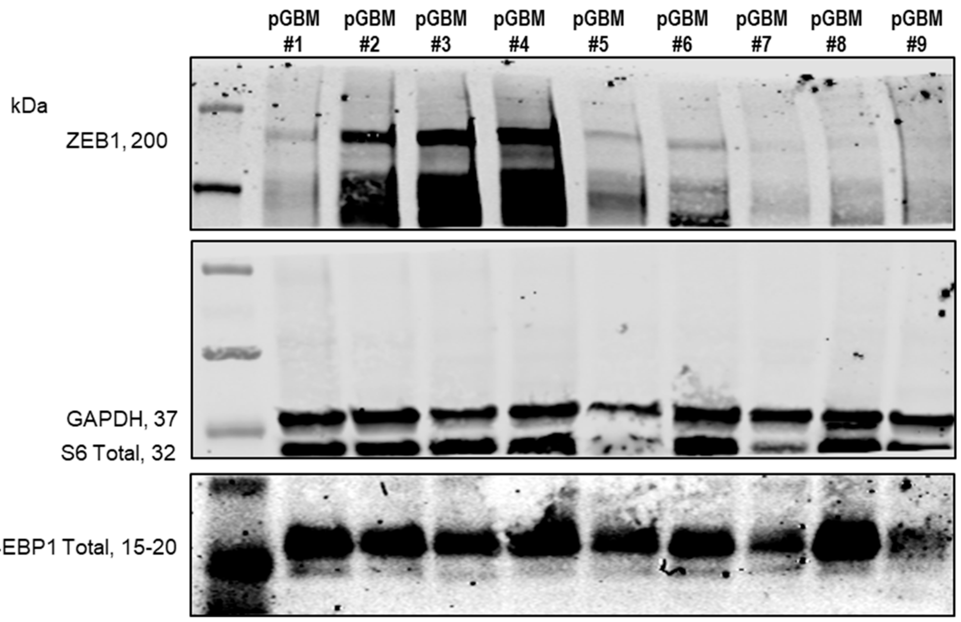

***ex vivo* tissue samples. Total Proteins.**

Part of the Figure 5(e)

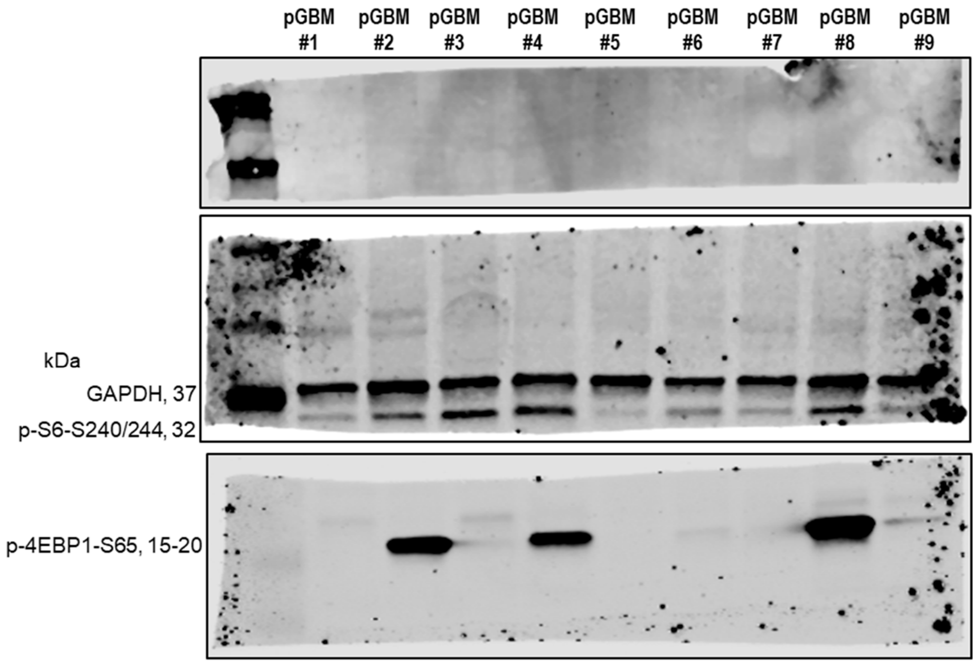

***ex vivo* tissue samples. Phosphorylated proteins.**
Part of the Figure 5(e)

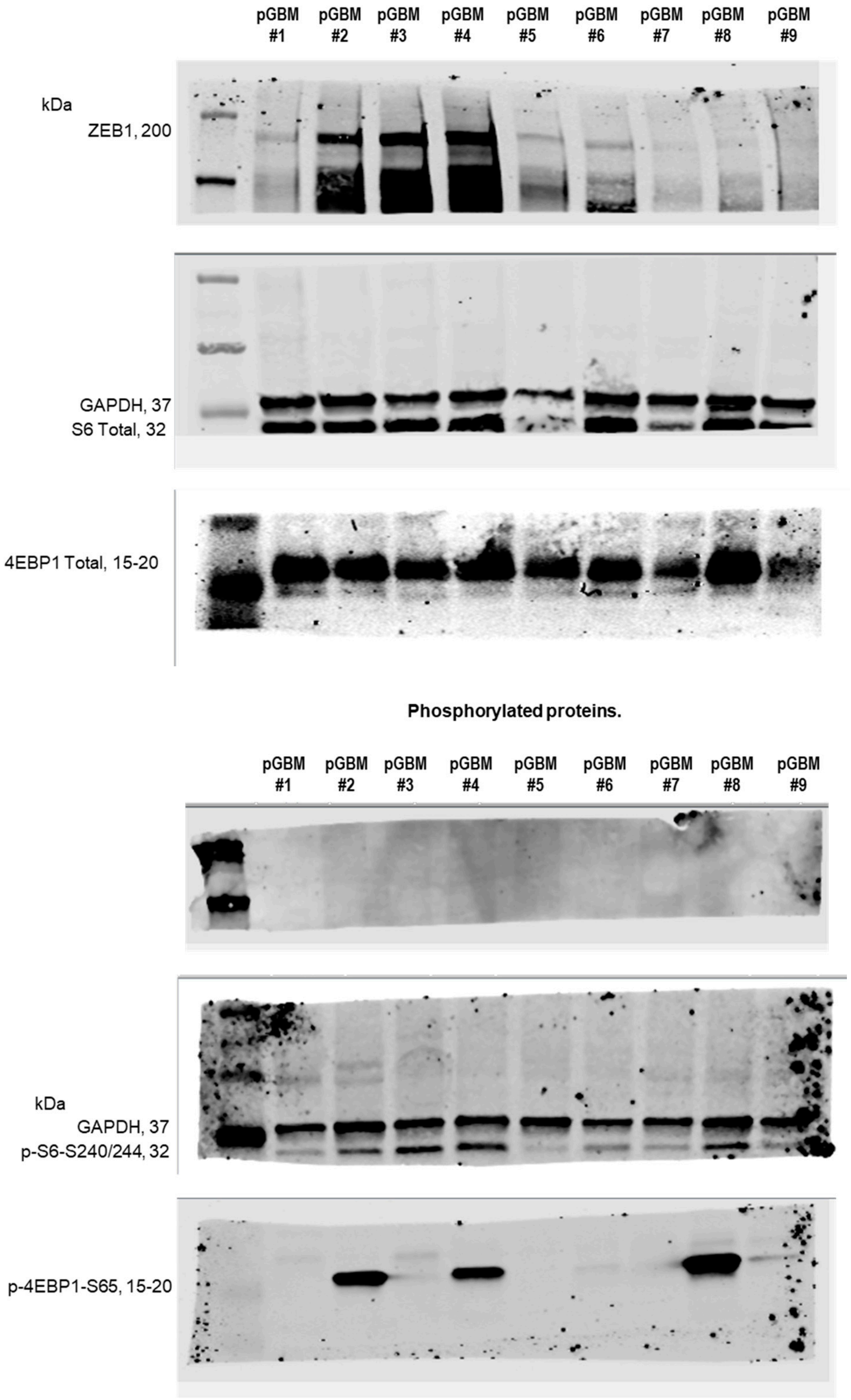
pGBM #1
pGBM #2
pGBM #3
pGBM #4
pGBM #5
pGBM #6
pGBM #7
pGBM #8
pGBM #9
kDa
ZEB1, 200
GAPDH, 37
S6 Total, 32
4EBP1 Total, 15-20
Phosphorylated proteins.
pGBM #1
pGBM #2
pGBM #3
pGBM #4
pGBM #5
pGBM #6
pGBM #7
pGBM #8
pGBM #9
kDa
GAPDH, 37
p-S6-S240/244, 32
p-4EBP1-S65, 15-20

JHH520

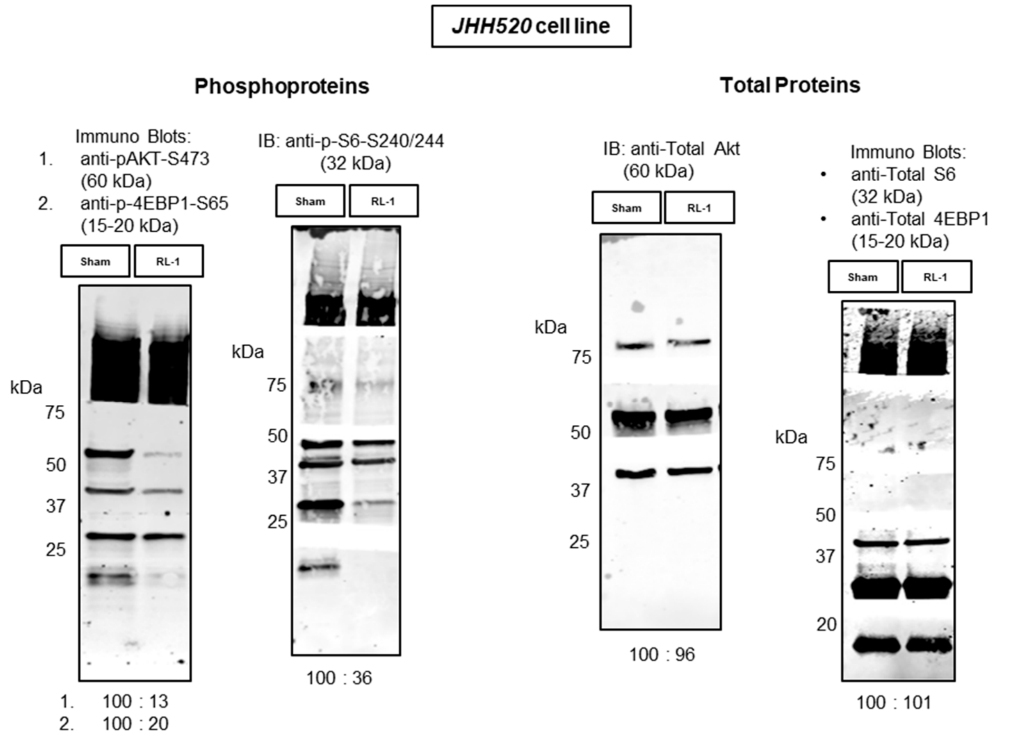

Figure 2a-b. RL1 effect on mTOR signaling protein marker expression.

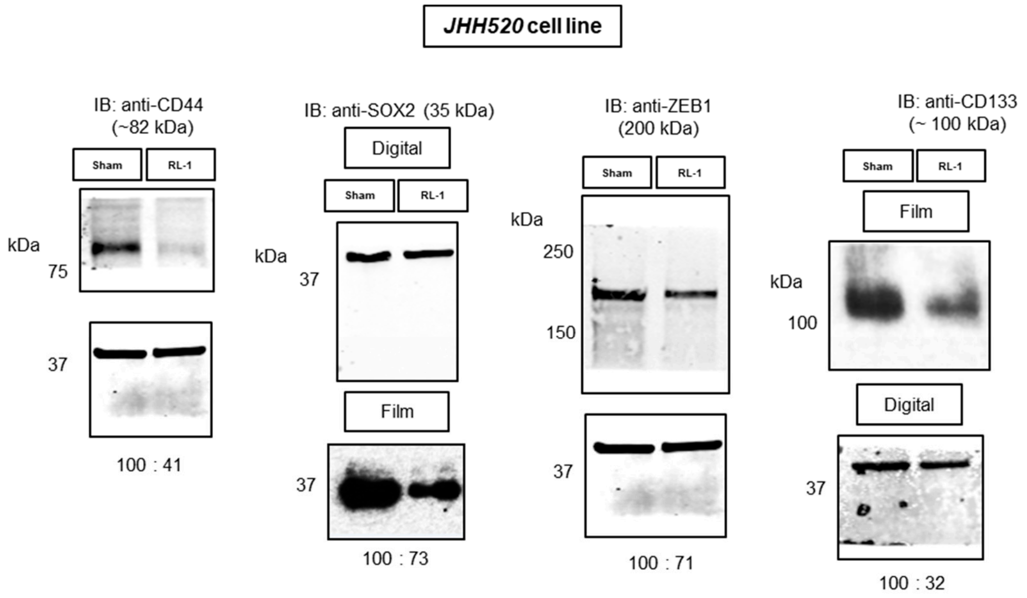

Figure 4a. RL1 effect on stemness and EMT protein marker expression.

BTSC233

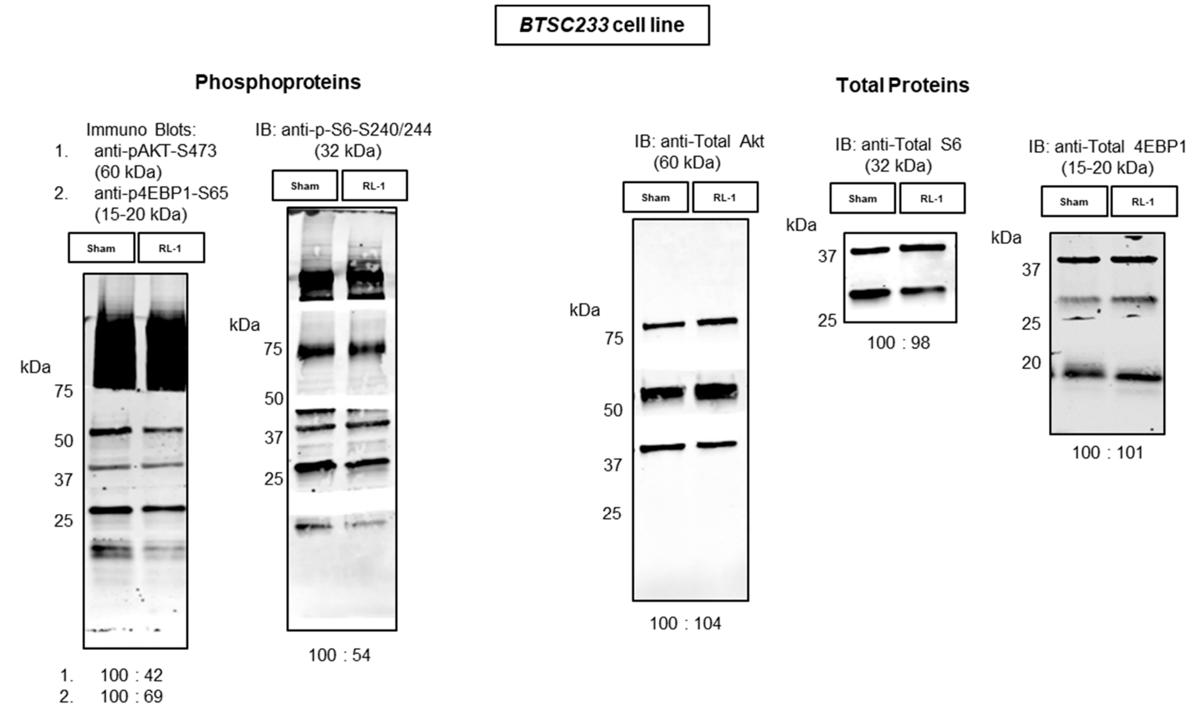

Figure 2a-b. RL1 effect on mTOR signaling protein marker expression.

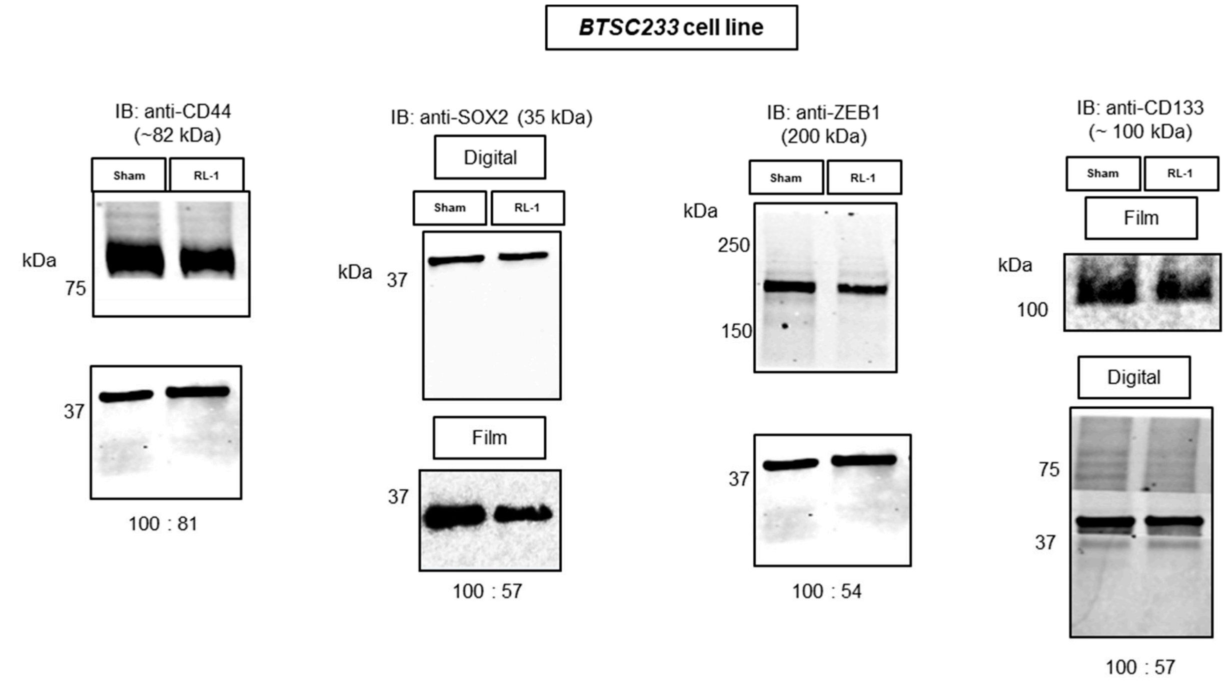

Figure 4a. RL1 effect on stemness and EMT protein marker expression.

NCH644

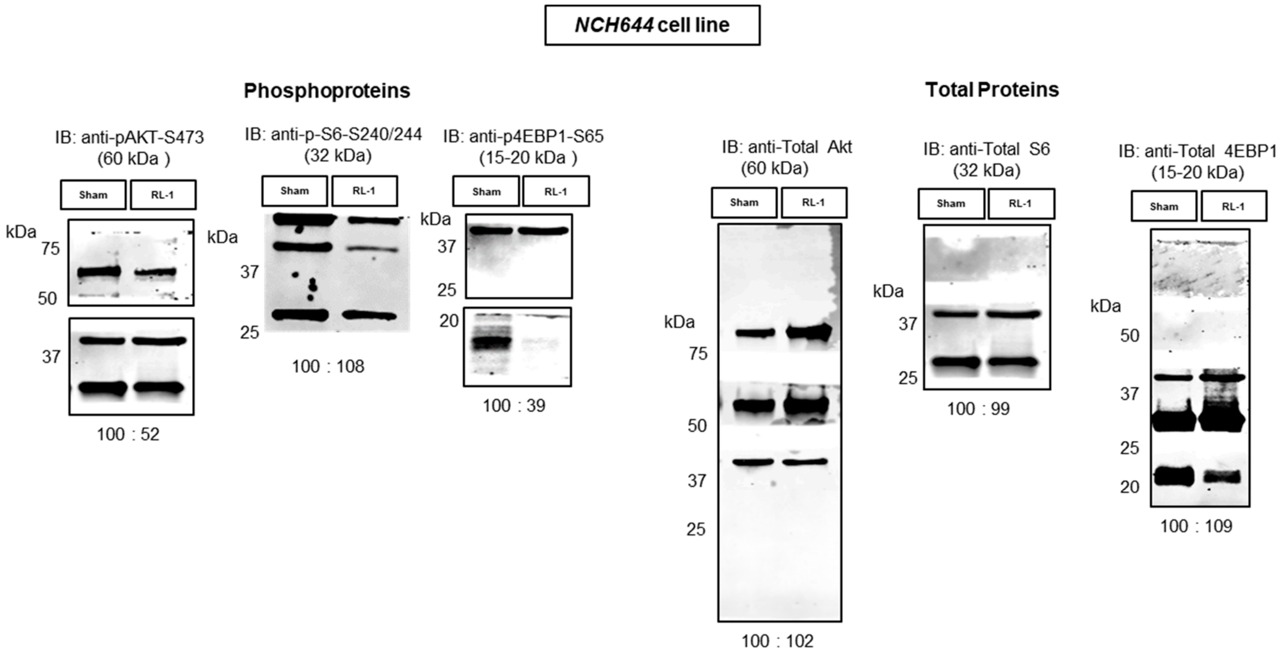

Figure 2a-b. RL1 effect on mTOR signaling protein marker expression.

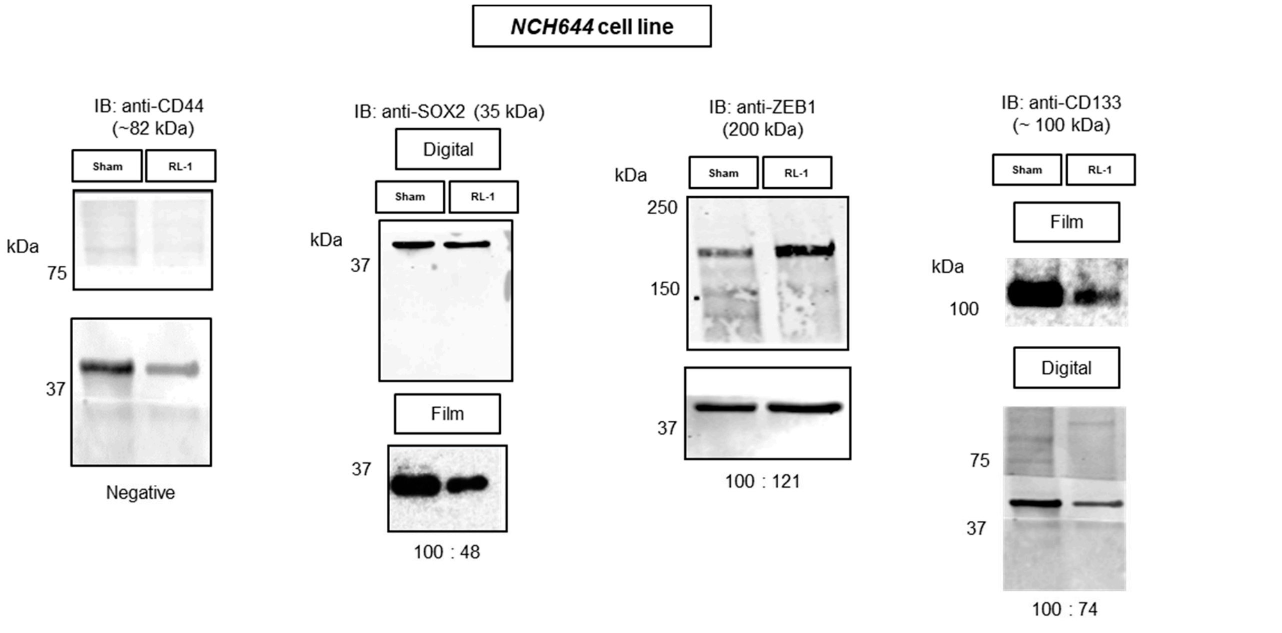

Figure 4a. RL1 effect on stemness and EMT protein marker expression.

Figure S2. Western blots membranes.

Relevant Notes: All bands were scanned digitally except otherwise written. Separate images were membranes that were cut physically before processing. Immuno Blots for the Screening of Figure 1b, were visually analysed without quantification before testing the drug (RL1). Quantified upon demand. Molecular weight estimation markers, performed with Precision Plus Protein ™ Dual Color Standards, 500 µl # 1610374.

Table S1. Western blot antibody information.

Antibody	MW (kDa)	Brand	Catalog Number	Concentration
**p-4EBP1-S65**	15-20	Cell Signaling Technology, Danvers, MA, USA	#9451	1:500
**Total 4EBP1**	15-20		#9644	1:1000
**p-S6-S240/244**	32		#2215	1:2000
**Total S6**	32		#2217	1:2000
**p-Akt-S473**	60		#9271	1:250
**Total Akt**	60		#4691	1:1000
**Sox2**	35		#L1D6A2	1:1000
**β-Actin**	42		#4970	1:5000
**CD44**	~82		#3570	1:100
**ZEB1**	200	Sigma	#HPA027524	1:2000
**CD133**	~100	Miltenyi, Germany	#W6B3C1	1:100
**GAPDH**	~37	Proteintech	#60004-1-Ig	1:10000

© 2020 by the author. Licensee MDPI, Basel, Switzerland. This article is an open access article distributed under the terms and conditions of the Creative Commons Attribution (CC BY) license (http://creativecommons.org/licenses/by/4.0/).
